# A Preventive and Control Strategy for COVID-19 Infection: An Experience From a Third-Tier Chinese City

**DOI:** 10.3389/fpubh.2020.562024

**Published:** 2020-10-15

**Authors:** Shushan Fan, Min Wu, Shengjun Ma, Shouguo Zhao

**Affiliations:** ^1^Department of Healthcare Associated Infection Control, Liaocheng People's Hospital, Liaocheng, China; ^2^Department of Cardiac Surgery, Liaocheng People's Hospital, Liaocheng, China; ^3^Department of Urology, Liaocheng People's Hospital, Liaocheng, China

**Keywords:** COVID-19, prevention and control, first level response, economy, strategy

## Abstract

COVID-19 is a rapidly spreading infectious disease that has led to a global pandemic. This study describes a novel strategy for preventing and controlling COVID-19 infection in the third-tier city of Liaocheng, China. The prevention and control measurements included city-wide orders to close workspaces, sanitize essential workspaces, quarantine individuals with a travel history to an epidemic area, and issue emergency medical responses to quarantine and treat COVID-19 patients using all necessary technologies, personnel, and resources. As a result, there were only 38 diagnosed COVID-19 cases in Liaocheng since the pandemic began in China in late 2019, including in the metropolitan area and six suburban counties, accounting for more than 6.39 millions residents living in a 8,715 km^2^ area. There was no COVID-19-related fatality and no healthcare professional inter-transmission as of June 25, 2020. The strategies of this third-tier Chinese city provide useful insights into approaches to prevent and control COVID-19 spread in other Chinese cities and countries.

## Introduction

The coronavirus disease 2019 (COVID-19) is a rapidly spreading infectious disease that has led to global pandemic (1–4). On June 25, 2020, there have been more than 9.44 million confirmed COVID-19 cases and 483 thousand COVID-19-related deaths in the world (5). Epidemiologically, COVID-19 is mainly spread by inhalation of droplets or fomites. Small droplets produced by an infected person with or without any symptoms can be aerosolized through coughing, sneezing, talking, or singing and then inhaled by persons in close contact. Furthermore, poorly ventilated environments leads increase potential of transmission (6, 7). In the clinic, the most common symptom of COVID-19 is fever, followed by cough, loss of appetite, fatigue, shortness of breath, sputum, and muscle and joint pains (8, 9), while other symptoms, like nausea, vomiting, or diarrhea can also occur (10, 11); common flu symptoms, such as sneezing, runny nose, sore throat, and skin lesions, are less common in COVID-19 patients [World Health Organization (WHO)]. Report of the WHO-China Joint Mission on Coronavirus Disease 2019 (COVID-19) on February 16–24, 2020 (https://www.who.int/docs/default-source/coronaviruse/who-china-joint-mission-on-covid-19-final-report.pdf). As there are no curable treatment options currently available, COVID-19 patients often suffer from complications including pneumonia, acute respiratory distress syndrome (ARDS), multi-organ failure, septic shock, heart failure, blood coagulation, liver injury, seizure, stroke, encephalitis, and even death (12–17). Thus, novel and effective preventive and control strategies for COVID-19 infection are crucial to ending this ongoing global pandemic. Here, we describe our experiences and implementation of strategies to control and prevent COVID-19 in a third-tier Chinese city with a population of 6.39 million residents. Our findings will hopefully provide insight for preventing and controlling the spread of COVID-19 in other Chinese cities and countries.

## Methods and Strategies

### Liaocheng Statistics and Study Population

Liaocheng is a third-tier Chinese city that spans 8,715 km^2^ in Western Shandong province. As of April 7, 2020, there have been 83,157 confirmed cases, 1,042 imported cases, and 1,095 asymptomatic cases of COVID-19 in China. In Liaocheng, which has a population of 6.39 million, there have been only 38 confirmed cases without any related fatality. The population in Liaocheng includes ~2.39 million residents in the metropolitan area and 4 million residents in its suburban six counties. In 2007, Liaocheng was named one of the top 10 most livable cities in China by the Chinese Cities Brand Value Report (https://archive.is/20130410050946/http://eng.hnloudi.gov.cn/engld%5Caboutloudi/Loudicity/Loudihonor/2011/1_327/default.shtml). Economically, the total 2019 gross domestic product (GDP) of Liaocheng accounted for 225.98 billion RMB, a 3% increase from 2018, among which the industrial GDP contributed 49.8% to the total GDP (2019 Liaocheng city GDP data. https://baike.baidu.com/item/%E8%81%8A%E5%9F%8E/127583). Among Liaocheng's 9.39 million residents, there are ~3.3 million males and ~3.1 million females, and ~1.6 million (25.2%) are 0 and 17 years, old, ~3.7 million (57.1%) are 18 and 59 years old, and ~1 (17.6%), are over 60 years old (Liaocheng population data from Liaocheng city municipal government). Liaocheng has 552 hospitals and clinics with 26,280 beds and 28,900 medical staff employees (data from Liaocheng city municipal government).

### Strategies to Handle the COVID-19 Crisis in Liaocheng

#### Implementation of Prevention and Control Mechanisms and Strengthening Organizational Leadership During the COVID-19 Pandemic

To effectively control and prevent the COVID-19 pandemic, the municipal government of Liaocheng first formed a COVID-19 pandemic workgroup on January 22, 2020, that provided guidance on the prevention and control of COVID-19 infection. Thereafter, the workgroup established 36 fever clinics and designated 10 medical institutions to manage and treat patients with COVID-19. This workgroup was formed as an urgent response to complement the research efforts and achieve the objectives of controlling and preventing COVID-19 infection. Moreover, this workgroup followed the central government's directives and adapted them to local situations by developing health policies and measurements related to infection control. This workgroup also strengthened communications between departments and updated the relevant departments on COVID-19 infection rates.

#### First Responses

At the onset of the crisis, the Shandong provincial government immediately activated a Level 1 crisis response on January 24, 2020, and Liaocheng followed the same protocol on the day when the city reported its first case of COVID-19. However, this occurred during the Chinese New Year and there was an increase in travel and movement between regions, leading to an increase in consecutive COVID-19 transmissions and risk of urban to suburban area transmission. Thus, Liaocheng implemented the strictest infection control measurements to prevent further COVID-19 spread and infection. The detailed strategies were as follows: (1) The city ordered a stoppage of activities and workplace closure, including museums, stadiums, libraries, tourist attraction sites, parks, cinemas, cafes, public facilities, all other entertainment facilities, fitness centers, and public and private schools. Only supermarkets, hospitals, fresh food markets, petrol stations, infant accessory shops, and pharmacies were allowed to remain open. (2) All shopping malls were ventilated at an automated setting, sterilized every 4 h, and shortened operating hours (open from 10 a.m.−6 p.m.). (3). Fresh markets were ordered to stop all wildlife trade and close all live animal trade and handling (meat butchering and preparation) to prevent potential animal to human transmission. (4) All immigration and imported goods were scrutinized and quarantined as necessary (for immigrants, there was a mandatory 14 days stay at home or hotel quarantine). Since January 27, 2020, all Liaocheng automobiles were ordered to stop operation except those for transportation of basic necessities. Public transportation, such as buses, operated at reduced frequencies and all riders were ordered to wear facemasks and ensure their body temperatures were <37.3°C before boarding the vehicle. Social distancing was also implemented on all public transportation, and mobile payments were encouraged instead of cash payments. (5) Residents with a significant travel history were ordered to wear facemasks and regularly monitor their body temperatures. Such individuals were asked to stay home, isolate themselves in a room, and be subject to screening by medical professionals and monitoring by the local authorities. (6) Residents were ordered to stop visitation and eating out and instructed to avoid any crowded places, including hospitals, shopping malls, and fresh markets. All food and beverage services in the public areas were ordered to stop and all funerals and weddings were scaled down to reduce large congregations of people. (7) Residents were highly encouraged to use personal protection equipment, such as facemasks, and adhere to good hygiene practices, especially using alcohol-based hand rubs or hand washing solutions. However, individuals with a fever or respiratory symptoms who were seeking medical attention were required to wear a facemask and avoid self-medicating during this period of the pandemic. (8) School reopening was postponed and online learning was implemented. Graduating students were given priority to return to school and the timing of their classes was staggered when feasible.

#### Prevention and Control of COVID-19 Infection in Urban and Suburban Areas

According to the Shandong provincial COVID-19 infection control plan, various measurements were implemented to further control the possible spread of COVID-19 between the urban and suburban regions. All districts within Liaocheng were ordered to close their borders (boundaries) and instructed to report individuals with a travel history. Entry and exit permits were issued for the individual district, facemasks were supplied to the local residents, and social gatherings were reduced or limited. In the suburban areas, all roads within the villages were closed and temperature measurements were instituted to prevent importation of COVID-19 cases. All villages were also instructed to reduce commuting and encouraged to stay home to reduce the risk of COVID-19 infection.

#### Control of COVID-19 Infection in Hospital and Healthcare Institutions

Before COVID-19 was officially regulated as an infectious disease, protection and prevention measurements among the medical staff employees were low, resulting in potential increases in cross-contamination and infection among the medical staff and patients. After COVID-19 was categorized as a deadly infectious disease with a greater risk than Severe Acute Respiratory Syndrome (SARS), the risk of an outbreak in the healthcare setting was eminent (18). In China, there were 1,716 medical staffs reported to be infected with COVID-19 (data as of June 25, 2020), resulting in the local government of Liaocheng to implement various strategies to reduce the risk of COVID-19 infection. (1) Risk assessment was carried out at urban and suburban levels and the appropriate resources, such as staff and equipment, were allocated to manage COVID-19 infection risk. The guidelines of regular monitoring and updates of COVID-19 infection were implemented to reduce the over or under control of COVID-19 infection. (2) Strict standards on infection control were implemented. For example, medical staff employees and relevant staff working in the fever clinics, isolation rooms, and laboratories were trained and tested on the use of personal protective equipment and all medical staff employees were required to wear facemasks and practice good hygiene when entering the facilities. The healthcare institutions were required to ventilate, sterilize, and differentiate the various routes for patients and medical staff to reduce cross-contamination in their facilities. Medical staff employees with direct contact of patient blood, body fluid, secretions, or aerosolizing procedures were required to wear the personal protective equipment, including facemasks, protective goggles or shields, and gowns. (3) All operations at the healthcare institutions were modified. For instance, patients were urged to schedule their appointments in advance to reduce their waiting time, and all patients had their temperatures measured before entering the hospitals. If a patient had a fever while in the healthcare facility, they would be sent to an isolation room and subjected to diagnosis and treatment procedures. (4) Acute respiratory wards were instituted to house and isolate patients at risk of COVID-19 infection. Further imaging scans, respiratory swabs, and blood tests were instituted to exclude COVID-19 infection before transferring any patient to the general ward. The healthcare staff movements were controlled via different access cards, and a non-visitation policy was instituted for the relatives or patients' next of kin. (5) Active surveillance of all medical staff was instituted. For example, all medical staff employees were required to periodically report their health status and temperature. The emergency department, laboratories, radiation oncology, operating theaters, and endoscopic center were monitored to reduce the spread of COVID-19 among staff. (6) Specific precautions were also taken for certain patients. For example, special arrangements were made for patients who needed emergency surgery, suffered acute myocardial infarction or cerebrovascular complication, or received periodic chemoradiotherapy treatments. All other patients who were usually in the outpatient setting were encouraged to delay their appointments while extending their prescriptions or having their consultations using telemedicine services.

#### Traditional Chinese Medicine (TCM) to Control COVID-19 Infection

Seven Chinese Medicine experts formulated a novel regimen using TCM to aid the management of patients with COVID-19 infection. Herbal soup was prepared for all first-line medical staff, policemen, widowed elderlies, and other vulnerable individuals in an effort to improve their immunity and decrease the risk of COVID-19 infection.

#### Novel Technologies to Control COVID-19 Infection

Multiple technology platforms were utilized to help fight the COVID-19 pandemic. (1) Telemedicine and telephone conversations were used to manage and maintain patient appointments, which helped to reduce patients coming into the medical facilities and to prevent cross-contamination and risk of COVID-19 infection. The popular Chinese smartphone application WeChat, which functions similarly to Skype and Facebook; was used among 36 medical organizations in Liaocheng to provide free real time online consultation services for patients with a fever, chronic diseases, or common ailments. Physicians were able to either diagnose symptoms online or refer patients to a medical facility as needed, which significantly reduced the waiting time and potential cross-infection. (2) Liaocheng created a common hotline operated by healthcare specialists to discuss and promote health knowledge. Liaocheng residents could use the hotline to receive psychological consultation to improve mental health during the COVID-19 pandemic. (3) Working at home was widely encouraged when feasible. Meeting in person was discouraged and telecommuting was advised.

#### Media-Related Health Wellness to Control COVID-19 Infection

Television, radio, and the Internet were efficiently utilized to spread news and health wellness advice to control COVID-19 infection. Through these platforms, medical and epidemiologist experts explained current information and created more supportive messages for the general population and local residents. This resulted in more residents taking charge of their health using the latest COVID-19 information.

## Results

### Control of COVID-19 Infection in Liaocheng

As of March 1, 2020, there were only 38 confirmed COVID-19 cases with no reported deaths in Liaocheng. On March 7, 2020, Shandong provincial government had re-classified Liaocheng from a Level 1 risk response area to a Level 2 risk response area. In Level 2, all normal life activities can be phased back in, including the use of public bus transportation for short and long distance commuters. On March 10, 2020, Liaocheng was further categorized as a low risk area of COVID-19 infection. As of the time of this study's publication, there has been no report of cross infection or contamination cases in the healthcare institutions and no infection among patients and the medical staff in Liaocheng.

### Characteristics of Patients With COVID-19 Infection

Detailed information of the 38 patients with COVID-19 infection is presented in Table 1. Specifically, the mean age of the patients was 43.17 ± 17.9 years (ranged between 6 months and 77 years) and included 18 males and 20 females. The underlying medical conditions were fever and cough and treatments included thymosin, oxygen therapy, and TCM. One patient stayed in the intensive care unit for 7 days with plasma exchange (PE) and high-flow nasal cannula (HFNC) supportive care (Table 1).

**Table 1:** Clinical characteristics of the COVID-19 patients in Liaocheng (*n* = 38).

**Characteristics and treatment**	**All patients *n* (%)**
**Age** (yrs.)	43.17 ± 17.9
**Gender**	
Male	18 (47.37)
Female	20 (52.63)
**The underlying diseases**	
Any	9 (23.68)
Hypertension	3 (7.89)
Coronary heart disease	3 (7.89)
Diabetes	2 (5.26)
Pulmonary interstitial fibrosis	1 (2.63)
Cirrhosis, liver cancer	1 (2.63)
**Epidemiological history**	
Contact with wild	0 (0)
Wuhan sojourn	6 (15.79)
Contact with a diagnosed patient or workplace	30 (78.95)
Not available	2 (5.26)
**Symptoms**	
Asymptomatic	8 (21.05)
Fever	23 (60.53)
≤ 38.0°C	20 (86.96)
>38.0°C	3 (13.04)
Chills	1 (2.63)
Fatigue	9 (23.68)
Headache	3 (7.89)
Nasal congestion	3 (7.89)
Sore throat	8 (21.05)
Cough	25 (65.79)
Hemoptysis	1 (2.63)
Shortness of breath	12 (31.58)
Vomiting or diarrhea	8 (21.05)
Pain in a muscle or joint	2 (5.26)
**Disease severity**	
Mild	6 (15.79)
Moderate	30 (78.95)
Severe	1 (2.63)
Critical	1 (2.63)
**Treatment**	
Antibiotics	27 (71.05)
Antifungal drugs	1 (2.63)
Antiviral drugs	37 (97.37)
Glucocorticoids	8 (21.05)
Albumin	12 (31.58)
Immunoglobulin	7 (18.42)
Thymosin	24 (63.16)
Oxygen therapy	15 (39.47)
Common	13 (34.21)
HFNC	2 (5.26)
PE	1 (2.63)
TCM	37 (97.37)

*HFNC, high-flow nasal cannula; PE, plasma exchange; TCM, traditional Chinese medicine*.

### Effectiveness of the Preventive and Control Strategies in Liaocheng

Because of the successful implementation and measurements to control COVID-19 infection in Liaocheng, the city has currently started to revive its economy, despite remaining in a critical period of the COVID-19 pandemic. As the number of COVID-19 cases continues to rise in other parts of China and the world, there has been no single COVIOD-19 infection case reported in Liaocheng as of June 25, 2020. We acknowledge that Liaocheng is still at risk of COVID-19 transmission and infection, and that newly developed asymptomatic cases and transmission could challenge our ability to prevent and control the COVID-19 pandemic.

### Lessons Learned From Liaocheng's Prevention and Control Strategies

During the past 6 months of the COVID-19 pandemic in Liaocheng, we successfully implemented various strategies and measurements to prevent and control COVID-19 infection. Our strategies and measurements to timely and effectively reduce COVID-19 in our community were successful. These prioritized initiative included: (1) issuing city-wide orders to close workspaces or reduce operation hours for essential facilities, like the public water, gas, or telecommunication facilities, medical staff, and supermarkets; (2) enforcing social distancing; (3) enforcing body temperature checks and DNA tests, when available; (4) enforcing the use of facemasks; and (5) quarantining and isolating individuals with a travel history to epidemic areas and who present with COVID-19-like symptoms.

## Discussion

In the current study, we summarized our implementation of various strategies and measurements to prevent and control COVID-19 infection in Liaocheng, a third-tier Chinese city. Our data showed that there were only 38 confirmed COVID-19 cases with no deaths in Liaocheng since the COVID-19 pandemic began in late 2019 through June 25, 2020. Our successful strategies and measurements led the Shandong provincial government to down-classify of our city on March 7, 2020 to a Level 2 risk response area and on March 10, 2020 to a low risk response area. At the time of this publication, our city has had no new cases or cross infection or contamination cases among patients and the medical staff. In conclusion, we implemented a successful model that could be emulated by similar-size cities worldwide to prevent and control the spread of COVID-19.

Among the 38 patients in our current study, eight had no symptoms or signs of infection, 20 presented with a low fever ( ≤ 38°C), and only three presented with a high fever. These clinical characteristics differed from regular signs of respiratory system infections, such as the common cold or flu. Among the 38 patients, 37 received TCM treatments and showed good responses. The TMCs included jin hua qing gan granules, lian hua qing wen capsules, and other prescriptions issued by TMC doctors. Because of the orders issued by the city government, Liaocheng successfully controlled the COVID-19 pandemic; however, globally the pandemic is far from contained, and we will continue to reinforce and follow the government orders and regulations to prevent and control another possible COVID-19 epidemic in our city.

During the past 6 months of the COVID-19 epidemic in China and in Liaocheng specifically, our residents followed the orders issued by all levels of government, which essentially prevented and controlled the COVID-19 epidemic in our city of 6.3 million people. The lessons learned from our response include the following: (1) Issue city-wide orders to close workspaces or reduce operation hours for essential facilities, like public water, gas, or telecommunication facilities, medical staff, and supermarkets. (2) Enforce social distancing. (3) Enforce body temperature checks and DNA testing, when feasible. (4) Everyone wears a facemask. (5) Quarantine and isolate individuals with a travel history to an epidemic area and who have COVID-19-like symptoms. In our approach, any individual who did not follow these orders could be arrested or prosecuted. In addition, if there was a diagnosed COVID-19 case in a building or community, that building or community was isolated (no one could enter or leave). These measurements were necessary in helping us to prevent and control COVID-19 infection. It is important to note that this report only provides insight into the prevention and control, but not into treatment strategies. Since our study only included 38 patients who did not have severe symptoms, our cohort size was too small to determine efficacy of TMC for treating COVID-19.

## Data Availability Statement

All datasets generated for this study are included in the article.

## Author Contributions

SF and MW are the principal investigators of this study. SF wrote the first draft of the manuscript. All authors conceived the study, contributed to the manuscript revision, read, and approved the submitted version.

## Conflict of Interest

The authors declare that the research was conducted in the absence of any commercial or financial relationships that could be construed as a potential conflict of interest.

## References

[1] Kannan S Shaik Syed Ali P Sheeza A Hemalatha K COVID-19 (novel coronavirus 2019) - recent trends. Eur Rev Med Pharmacol Sci. (2020) 24:2006−11. 10.26355/eurrev_202002_20378 32141569

[2] Zhai P Ding Y Wu X Long J Zhong Y Li Y. The epidemiology, diagnosis and treatment of COVID-19. Int J Antimicrob Agents. (2020) 55:105955. 10.1016/j.ijantimicag.2020.105955 32234468 PMC7138178

[3] Ge H Wang X Yuan X Xiao G Wang C Deng T. The epidemiology and clinical information about COVID-19. Eur J Clin Microbiol Infect Dis. (2020) 39:1011–9. 10.1007/s10096-020-03874-z 32291542 PMC7154215

[4] Sohrabi C Alsafi Z O'Neill N Khan M Kerwan A Al-Jabir A. World Health Organization declares global emergency: a review of the 2019 novel coronavirus (COVID-19). Int J Surg. (2020) 76:71–6. 10.1016/j.ijsu.2020.02.034 32112977 PMC7105032

[5] Ramphul K Mejias SG. Coronavirus disease: a review of a new threat to public health. Cureus. (2020) 12:e7276. 10.7759/cureus.7276 32300496 PMC7158591

[6] Ding J Fu H Liu Y Gao J Li Z Zhao X. Prevention and control measures in radiology department for COVID-19. Eur Radiol. (2020) 30:3603–8. 10.1007/s00330-020-06850-5 32300968 PMC7160611

[7] Hamner L Dubbel P Capron I Ross A Jordan A Lee J. High SARS-CoV-2 attack rate following exposure at a choir practice - Skagit County, Washington, March 2020. MMWR Morb Mortal Wkly Rep. (2020) 69:606–10. 10.15585/mmwr.mm6919e6 32407303

[8] Chen N Zhou M Dong X Qu J Gong F Han Y. Epidemiological and clinical characteristics of 99 cases of 2019 novel coronavirus pneumonia in Wuhan, China: a descriptive study. Lancet. (2020) 395:507–13. 10.1016/S0140-6736(20)30211-7 32007143 PMC7135076

[9] Grant MC Geoghegan L Arbyn M Mohammed Z McGuinness L Clarke EL. The prevalence of symptoms in 24,410 adults infected by the novel coronavirus (SARS-CoV-2; COVID-19): a systematic review and meta-analysis of 148 studies from 9 countries. PLoS ONE. (2020) 15:e0234765. 10.1371/journal.pone.0234765 32574165 PMC7310678

[10] Huang C Wang Y Li X Ren L Zhao J Hu Y. Clinical features of patients infected with 2019 novel coronavirus in Wuhan, China. Lancet. (2020) 395:497–506. 10.1016/S0140-6736(20)30183-5 31986264 PMC7159299

[11] Lai CC Shih TP Ko WC Tang HJ Hsueh PR. Severe acute respiratory syndrome coronavirus 2 (SARS-CoV-2) and coronavirus disease-2019 (COVID-19): the epidemic and the challenges. Int J Antimicrob Agents. (2020) 55:105924. 10.1016/j.ijantimicag.2020.105924 32081636 PMC7127800

[12] Murthy S Gomersall CD Fowler RA. Care for critically ill patients with COVID-19. JAMA. (2020) 323:1499. 10.1001/jama.2020.3633 32159735

[13] Heymann DL Shindo N. COVID-19: what is next for public health? Lancet. (2020) 395:542–5. 10.1016/S0140-6736(20)30374-3 32061313 PMC7138015

[14] Long B Brady WJ Koyfman A Gottlieb M Cardiovascular complications in COVID-19. Am J Emerg Med. (2020) 38:1504–7. 10.1016/j.ajem.2020.04.048 32317203 PMC7165109

[15] Xu L Liu J Lu M Yang D Zheng X. Liver injury during highly pathogenic human coronavirus infections. Liver Int. (2020) 40:998–1004. 10.1111/liv.14435 32170806 PMC7228361

[16] Sanders JM Monogue ML Jodlowski TZ Cutrell JB. Pharmacologic treatments for coronavirus disease 2019 (COVID-19): a review. JAMA. (2020) 323:1824–36. 10.1001/jama.2020.6019 32282022

[17] Carod-Artal FJ. Neurological complications of coronavirus and COVID-19. Rev Neurol. (2020) 70:311–22. 10.33588/rn.7009.2020179 32329044

[18] Hui DS E IA Madani TA Ntoumi F Kock R Dar O. The continuing 2019-nCoV epidemic threat of novel coronaviruses to global health - the latest 2019 novel coronavirus outbreak in Wuhan, China. Int J Infect Dis. (2020) 91:264–6. 10.1016/j.ijid.2020.01.009 31953166 PMC7128332

